# High-Fat or High-Carbohydrate Meal—Does It Affect the Metabolism of Men with Excess Body Weight?

**DOI:** 10.3390/nu14142876

**Published:** 2022-07-13

**Authors:** Lucyna Ostrowska, Joanna Smarkusz-Zarzecka, Anna Muszyńska, Edyta Adamska-Patruno, Maria Górska, Adam Krętowski

**Affiliations:** 1 Department of Dietetics and Clinical Nutrition, Medical University of Bialystok, ul. Mieszka I 4B, 15-054 Bialystok, Poland; lucyna.ostrowska@umb.edu.pl (L.O.); anna.muszynska1@umb.edu.pl (A.M.); 2 Clinical Research Support Centre, Medical University of Bialystok, ul. M. Sklodowskiej-Curie 24a, 15-276 Bialystok, Poland; edyta.adamska-patruno@umb.edu.pl; 3 Department of Endocrinology, Diabetology and Internal Medicine, Medical University of Bialystok, ul. M. Sklodowskiej-Curie 24a, 15-276 Bialystok, Poland; mgorska25@wp.pl (M.G.); adamkretowski@wp.pl (A.K.)

**Keywords:** high-fat meal, high-carbohydrate meal, obesity, energy expenditure, oxidation, glucose, insulin, triglycerides, homocysteine

## Abstract

Excessive adipose tissue in the body may lead to adverse health effects, carbohydrate and lipid metabolism disorders, and cardiovascular diseases. The aim of this study was to analyze the effect of a standardized high-fat meal (HF) on changes in energy expenditure and changes in the oxidation of energy substrates as well as the concentration of glucose, insulin, triglycerides and homocysteine in blood serum in relation to a standardized high-carbohydrate (non-fat, HC) meal in men with different nutritional status. In this study, 26 men (aged 19–60) without carbohydrate disorders (study group G_S_ = 13 overweight/obese; control group G_C_ = 13 normal body weight) were examined. It was observed that following a high-fat or high-carbohydrate meal, men with excessive body weight metabolized the main nutrients differently than men with normal body weight, and postprandial insulin secretion was also different (even without any significant differences in glucose concentrations). Overweight/obesity, which is in itself a risk factor for cardiovascular disease, contributes to an increase in the concentration of other risk factors, such as the concentration of homocysteine and triglycerides, which is referred to as cardiometabolic risk. Consumption of a high-fat meal increased the number of potential risk factors for cardiovascular disease (homocysteine and triglycerides) compared to a high-carbohydrate meal.

## 1. Introduction

Obesity has become a global problem. According to the latest data from the World Health Organization (WHO), the prevalence of obesity in the world almost tripled between 1975 and 2016. In 2016, over 1.9 billion adults had excess body weight, and 650 million of them were obese [1]. Obesity has been defined as a chronic disease with no tendency to resolve spontaneously and with a tendency to relapse [2]. Excessive accumulation of adipose tissue in the body, which exceeds physiological needs and adaptation abilities, can lead to adverse health effects, including carbohydrate and lipid metabolism disorders and cardiovascular disease (CVD) [3]. A new parameter in the assessment of carbohydrate and lipid homeostasis is postprandial dysmetabolism. Conventional risk factors for CVD are assessed in the fasting state. Postprandial dysmetabolism, however, is the postprandial state characterized by abnormally elevated levels of serum glucose and lipids, and is an independent risk factor for cardiovascular events [4]. Considering that most people eat fat-containing meals frequently during the day, the usual metabolism of lipids is postprandial since after fatty meal consumption, serum triglycerides increase within 1 h and remain elevated for 5–8 h. This contrasts with glucose metabolism, which displays transient elevation after a meal [5]. Elevated serum triglyceride and homocysteine levels are among the several risk factors for CVD [6]. In the treatment of obesity, pharmacological and non-pharmacological strategies are used. Caloric restriction and increased physical activity play a significant role in the non-pharmacological management of the disease. In clinical conditions, among the methods used to assess the effect of meals with different macronutrient content on energy expenditure and the oxidation of energy substrates is indirect calorimetry and the assessment of blood serum parameters. The method is based on the premise that the energy used by the body is obtained from the oxidation of nutrients (carbohydrates, proteins and fats) [7].

The broader focus of our research efforts is to evaluate potential differences in energy expenditure, glucose and lipid oxidation between men with different nutritional status and the impact of these variations on the further course of this disease in individuals with obesity [8]. Moreover, the limited number of scientific reports concerning the influence of the different nutritional composition of meals on energy expenditure and oxidation of nutritional substrates in the available literature encourages research in this area.

The aim of our study was to compare the effect of a standardized high-fat meal versus a standardized high-carbohydrate (non-fat) meal on changes in energy expenditure and the oxidation of energy substrates as well as the concentration of glucose, insulin, triglycerides and homocysteine in the blood serum of men with different nutritional status. We investigated the postprandial effect since direct and proportional associations between postprandial dysmetabolism and type 2 diabetes as well as cardiovascular diseases and events have been demonstrated [9,10].

## 2. Materials and Methods

This study was approved by the local Bioethics Committee of the Medical University of Bialystok, Poland (No. R-I-002/35/2009). This study is part of a larger project registered at clinicaltrials.gov as NCT03792685. In the present study, data from 26 individuals who met the inclusion criteria were analyzed. Inclusion criteria were: age 19–60 years and no carbohydrate metabolism disorders (to exclude people with carbohydrate disorders, a 75 g oral glucose tolerance test was performed). Exclusion criteria were: endocrine disorders, gastrointestinal diseases, renal/hepatic insufficiency, and other conditions that might have affected the final results, i.e., past bariatric and gastroenterological surgery, significant changes in body weight in the three months preceding this study, pharmacological treatment and use of other preparations, the influence of which on hormonal and metabolic changes in the body has not been documented or is not known. Only men were included in this study since the investigated parameters might have been characterized by sexual dimorphism, and therefore more confounding factors would have had to be considered in the analysis, such as hormones and menstrual cycle phases, which was not the aim of our study [11,12,13]. Men with a body mass index (BMI) ≥ 25.0 kg/m^2^ were included in the study group—G_S_ (overweight/obese; *n* = 13). Men with a BMI < 25.0 kg/m^2^ were included in the control group—G_C_ (normal body weight; *n* = 13). Participant characteristics are presented in Table 1.

Standardized, isocaloric meals with different nutritional composition were used to conduct the crossover study. The first was a high-fat (HF) meal (Calogen, Nutricia, Poland, 96% energy from fats, 4% from carbohydrates and 0% from proteins; energy value—−450 kcal) and the second was a high-carbohydrate (HC) meal (Nutridrink Fat Free, Nutricia, Poland, 89% energy from carbohydrates, 0% from fats and 11% from proteins; energy value −450 kcal). In order to obtain the energy value of both meals at the level of 450 kcal, a 300 mL portion of the HC meal and a 95 mL portion of the HF meal were used. Table 2 shows the composition of the HC and HF meals.

This study was conducted in two stages (two test meal visits). Subjects received the standardized HC or HF meals in random order, in a crossover design. Subjects arrived at our laboratory fasted between 6 and 10 a.m., and were asked to rest for 30 min in the supine position. Following that, cannulation of the antecubital vein was performed, and blood was collected for the determination of fasting glucose, insulin, triglyceride and homocysteine levels, and resting metabolism, fat and carbohydrate oxidation were assessed (30-min recordings). Then, the participants received a randomly assigned HC or HF meal (at room temperature) with a recommendation to consume it within 10 min. The subjects remained supine throughout the examination. Venous blood was drawn at 30, 60, 120, 180 and 240 min after HF or HC meal consumption to determine the concentration of glucose, insulin, triglycerides and homocysteine, while at 60, 120, 180 and 240 min after meal ingestion, energy expenditure was determined and glucose/lipid oxidation was assessed.

After 2 weeks, all participants received the second meal—HC or HF meal (those who had an HF meal during the first visit received an HC meal, and those who had ac HC meal on the first visit received an HF meal at room temperature)—and went through the same procedures as on the first visit.

### 2.1. Biochemical Analysis

The biochemical parameters were assessed at the Laboratory of the Department of Endocrinology, Diabetology and Internal Diseases, Medical University of Bialystok, Poland. Some of the parameters (glucose and triglycerides) were determined immediately after venous blood collection. The remainder of the biological material was stored in accordance with the recommendations of the manufacturers of the laboratory kits (−20 °C/−80 °C), and determinations were performed after the entire group of men had been tested. Serum glucose levels were measured by the hexokinase/glucose-6-phosphate dehydrogenase (G6PD) method using the Cobas c111 analyzer (Roche Diagnostics Ltd., Rotkreuz ZG, Switzerland). Serum insulin levels were assessed by the immunoradiometric method (RIA) with the use of INS-IRMA kits (DiaSource Immuno-Assays S.A., Ottignies-Louvain-la-Neuve, Belgium). The Wallac Wizard 1470 Automatic Gamma Counter (PerkinElmer, Life Science, Turku, Finland) was used for the determination of insulin. Serum triglycerides levels were determined by the enzymatic-colorimetric method according to Wahlefeld using the Cobas c111 analyzer (Roche Diagnostics Ltd., Rotkreuz ZG, Switzerland). Serum homocysteine levels were measured using the enzyme immunoassay (ELISA) method with the Human Homocysteine (Hcy) ELISA KIT (MyBioSource, Inc., San Diego, CA, USA).

### 2.2. Calorimetry Measurements

The assessment of resting metabolism, energy expenditure, the oxidation of fat and carbohydrates was conducted using the indirect calorimetry method with a metabolic monitor (Vmax Encore 29n System, Viasys HealthCare, Yorba Linda, CA, USA). All measurements during the indirect calorimetry test were performed with the participants lying down, in thermal comfort (room temperature within 21–25 °C), in a relaxed state, but with a recommendation not to fall asleep. Duration of a single recording of the test was approximately 30 min.

### 2.3. Statistical Analysi

Descriptive statistics were prepared by designating mean and standard deviation values for quantitative features. The studied group was taken as the classifying variable, and two levels of the classifying variable were determined: overweight/obese men (Gs) and normal body weight men (Gc). The choice of the statistical test depended on checking the following conditions: (1) homogeneity of variance in the groups and (2) normality of distribution of the dependent variable in the groups. If both conditions were met, one-way repeated-measures ANOVA was used, if not, the non-parametric Mann–Whitney U test was used. The area under the curve (AUC) of the values of the variables was calculated using the trapezoidal rule. The interrelationship between selected variables was obtained using the Spearman rank-order correlation coefficient (r). Statistical significance was set at *p* < 0.05. STATISTICA 13.3 (by Stat Soft, Cracow, Poland) was used for statistical analysis.

## 3. Results

In order to assess the effect of the investigated meals on the metabolic parameters in men with different nutritional status (without glucose disturbances), changes over the four-hour period in blood serum glucose, insulin, triglyceride and homocysteine concentrations following HF and HC meal ingestion were assessed, and the results are presented in Table 3.

### 3.1. Blood Glucose Concentration

**Figure 1:**
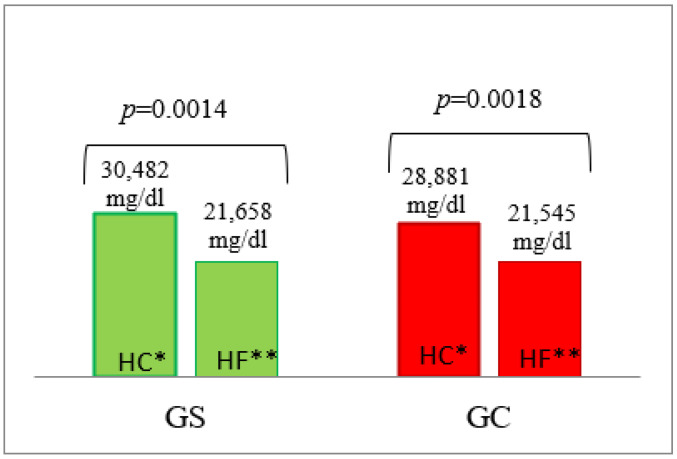
The AUC of glucose concentrations in the G_S_ and G_C_ groups after HC and HF meals. The results are presented as the mean values. * difference between the G_S_ (obese/overweight) and G_C_ (normal body weight) groups after HC meal (high-carbohydrate meal (*p* = 0.5726)); ** difference between the G_S_ and G_C_ groups after HF meal (high-fat meal (*p* = 0.7004)).

Serum glucose concentrations and the AUC of serum glucose concentrations following the HF and the HC meal were very similar in men from both groups (Table 3, and Supplementary Figure S1a,b). Glucose AUC values were, as expected, statistically significantly higher after the HC vs. the HF meal both in G_S_ (*p* = 0.0014) and G_C_ (*p* = 0.0018) groups (Figure 1).

There were no statistically significantly correlations between postprandial serum glucose concentrations, following either the HF or the HC meal, and BMI.

### 3.2. Serum Insulin Concentration

**Figure 2:**
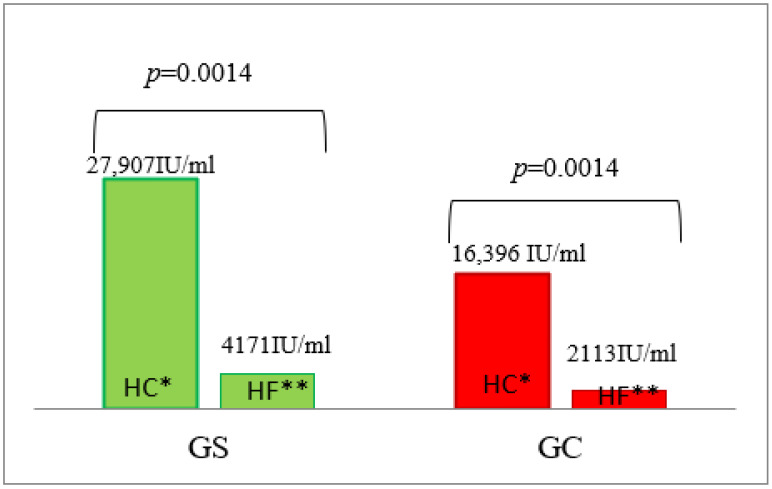
The AUC of insulin concentrations in the G_S_ and G_C_ groups after HC and HF meals. The results are presented as the mean values. * difference between the G_S_ (obese/overweight) and G_C_ (normal body weight) groups after HC meal (high-carbohydrate meal (*p* = 0.8979)); ** difference between the G_S_ and G_C_ groups after HF meal (high-fat meal (*p* = 0.0040)).

Despite the absence of carbohydrate metabolism disorders in the Gs group of men, fasting serum insulin concentrations were higher in this group (Table 3) compared to subjects from the control group. After the HF meal, serum insulin concentrations in the G_S_ group were significantly higher during the 30–180 min postprandial period compared to the control group (Table 3, Supplementary Figure S2a), which led to a significantly higher AUC of postprandial insulin concentrations in the G_S_ group compared to the control group (Table 3, Figure 2).

Surprisingly, no statistically significant differences in serum insulin concentrations were found between studied groups during the four-hour observation period following HC meal consumption, although higher, but not statistically significant, postprandial insulin levels were observed after HC meal ingestion at 30, 60 and 120 min in the G_S_ group (Table 3, Supplementary Figure S2b). However, as expected, the AUC of postprandial serum insulin concentrations was significantly higher following the HC vs. the HF meal, both in the G_S_ (*p* = 0.0014) and G_C_ (*p* = 0.0014) groups (Figure 2).

It was found that BMI was positively correlated with fasting insulin concentration (*p* = 0.0375; r = 0.4099). Statistically significant positive correlations were also observed between BMI and serum insulin concentration after HF meal ingestion at 120 (*p* = 0.0007; r = 0.6205) and 240 (*p* = 0.0386; r = 0.0386) minutes of the test. There were no significant correlations between serum insulin concentrations and BMI in either group after HC meal consumption.

### 3.3. Triglyceride Concentrations

**Figure 3:**
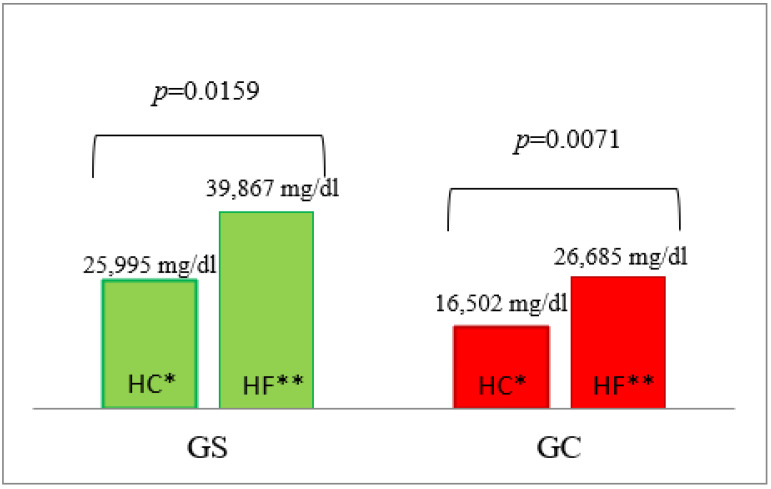
The AUC of triglyceride concentrations in the G_S_ and G_C_ groups after HC and HF meals. The results are presented as the mean values. * difference between the G_S_ (obese/overweight) and G_C_ (normal body weight) groups after HC meal (high-carbohydrate meal (*p* = 0.0274)); ** difference between the G_S_ and G_C_ groups after HF meal (high-fat meal (*p* = 0.0240)).

We observed that fasting triglyceride concentrations as well as concentrations after the HF meal (at 30, 120, 180 min of test) and the HC meal (30–180 min of the test) were significantly higher in the G_S_ group compared to the G_C_ group (Table 3, Supplementary Figure S3a,b). The high serum triglyceride concentrations observed in the G_S_ group were sustained for four hours after HF meal consumption, and resulted in higher AUC (G_S_: 39,867 mg/dL vs. G_C_: 26,685 mg/dL (*p* = 0.0240)), as shown in Figure 3. A statistically significantly higher AUC of triglyceride concentrations was also demonstrated in the G_S_ group vs. the G_C_ group after HC meal ingestion (*p* = 0.0274). Moreover, as we expected, significantly higher AUC values of triglycerides were observed in both groups: G_S_ (*p* = 0.0159) and G_C_ (*p* = 0.0071), following HF meal consumption in comparison to HC meal ingestion (Figure 3). Furthermore, a statistically significant positive correlation was demonstrated between BMI and the fasting serum concentration of triglycerides (*p* = 0.0142; r = 0.4749), and the concentrations at 120 (*p* = 0.0048; r = 0.5254) and 240 (*p* = 0.0427; r = 0.4002) minutes after the ingestion of the HC meal, which was not observed after HF meal consumption.

### 3.4. Homocysteine Concentrations

**Figure 4:**
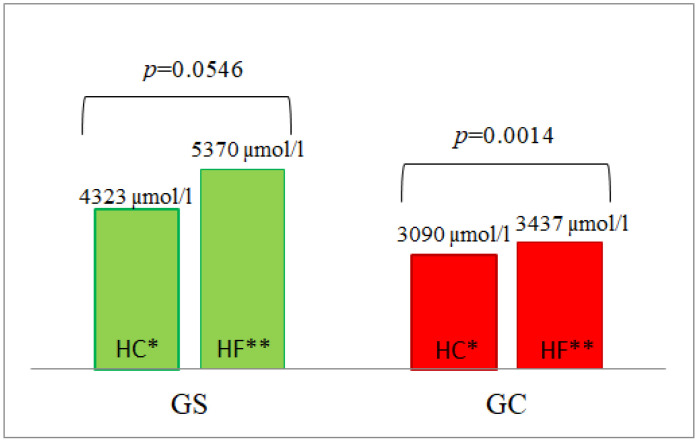
The AUC of homocysteine concentrations in the G_S_ and G_C_ groups after HC and HF meals. The results are presented as the mean values. * difference between the G_S_ (obese/overweight) and G_C_ (normal body weight) groups after HC meal (high-carbohydrate meal (*p* = 0.0138)); ** difference between the G_S_ and G_C_ groups after HF meal (high-fat meal (*p* = 0.0040)).

Statistically significantly (*p* = 0.0221) higher fasting homocysteine concentrations were found in the G_S_ group in comparison to the G_C_ group before HC meal ingestion. Higher serum homocysteine concentrations were also observed in the G_S_ group after consumption of both HF and HC meals (Table 3, Supplementary Figure S4a,b). A statistically significant difference (*p* = 0.0040) was found in the AUC of serum homocysteine postprandial concentrations following the HF meal between the groups. Furthermore, statistically significant differences (*p* = 0.0138) were observed in the AUC of postprandial homocysteine concentrations after the HC meal between the G_S_ and the G_C_ groups (Table 3, Figure 4).

There were statistically significant positive correlations between BMI and serum homocysteine concentrations following both the HC meal (fasting: *p* = 0.001; r = 0.6690 and during the second hour of the test *p* = 0.0010; r = 0.6069) and the HF meal (in the second: *p* = 0.0012; r = 0.5970 and fourth hour of the test *p* = 0.0054; r = 0.5286)

### 3.5. Indirect Calorimetry

In both studied groups, changes in energy expenditure, carbohydrate and fat oxidation after the HF meal, and then after the HC meal were assessed, as shown in Table 4. There were no statistically significant differences between the groups in energy expenditure, and glucose and fat oxidation, both in a fasted state and after the HF meal. However, after the HC meal, a statistically significant higher glucose oxidation level was demonstrated at 240 min after the ingestion of the meal in the Gs group.

#### 3.5.1. Energy Expenditure

**Figure 5:**
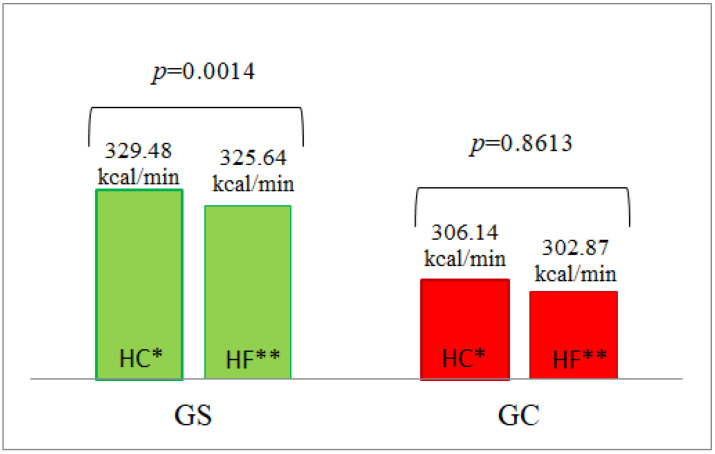
The AUC of energy expenditure in the G_S_ and G_C_ groups after HC and HF meals. The results are presented as the mean values. The results are presented as the mean values. * difference between the G_S_ (obese/overweight) and G_C_ (normal body weight) groups after HC meal (high-carbohydrate meal (*p* = 0.6444)); ** difference between the G_S_ and G_C_ groups after HF meal (high-fat meal (*p* = 0.3693)).

There were no statistically significant differences between the groups in energy expenditure both in a fasted state, and following HF and HC meal ingestion (Table 4, Supplementary Figure S5a,b). A statistically significant difference (*p* = 0.0014) was found in the AUC of energy expenditure after the HC meal vs. the HF meal in the G_S_ group (Figure 5).

#### 3.5.2. Glucose Oxidation

We did not observe any significant differences between the groups in glucose oxidation, except for higher glucose oxidation in the Gs group at 240 min after the ingestion of the HC meal (*p* = 0.0479) (Table 4, Supplementary Figure S6a,b).

**Figure 6:**
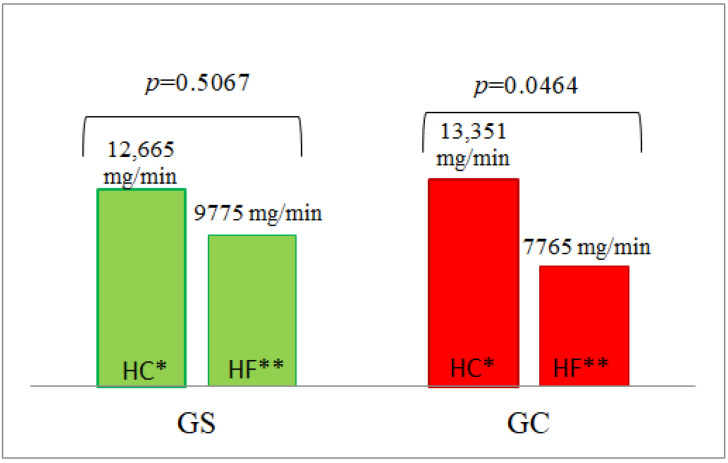
The AUC of glucose oxidation in the G_S_ and G_C_ groups after HC and HF meals. The results are presented as the mean values. * difference between the G_S_ (obese/overweight) and G_C_ (normal body weight) groups after HC meal (high-carbohydrate meal (*p* = 0.3558)); ** difference between the G_S_ and G_C_ groups after HF meal (high-fat meal (*p* = 0.2088)).

In the G_C_ group, the AUC of glucose oxidation after the HC meal was significantly higher than after the HF meal (*p* = 0.0464), which was not observed in the G_S_ group (Figure 6).

#### 3.5.3. Lipid Oxidation

**Figure 7:**
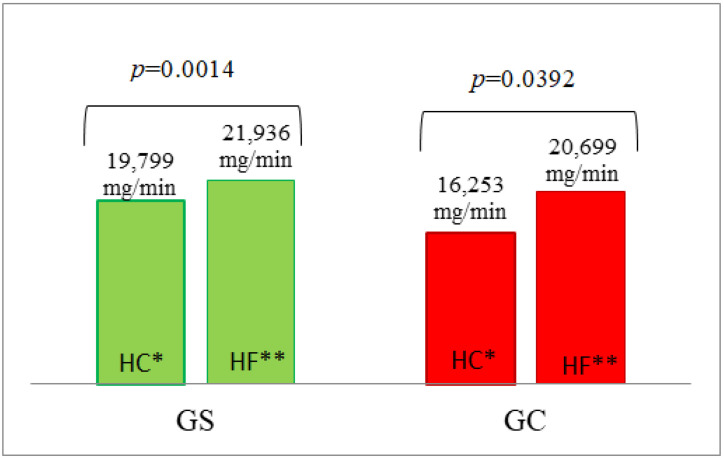
The AUC of lipid oxidation in the G_S_ and G_C_ groups after HC and HF meals. The results are presented as the mean values. * difference between the G_S_ (obese/overweight) and G_C_ (normal body weight) groups after HC meal (high-carbohydrate meal (*p* = 0.1909)); ** difference between the G_S_ and G_C_ groups after HF meal (high-fat meal (*p* = 1.000)).

Higher levels of fasting lipid oxidation were observed in the G_S_ group compared to the G_C_ group (Table 4, Supplementary Figure S7a,b). A significantly higher AUC of lipid oxidation was observed in both the G_S_ (*p* = 0.0014) and G_C_ (*p* = 0.0392) groups after HF vs. HC meal ingestion (Figure 7).

## 4. Discussion

Treatment of obesity can be based on non-pharmacological methods and supportive treatment with the use of pharmacotherapy. Lifestyle modification, which involves altering long-term habits of eating and physical activity, and maintaing the new behaviour allows for a gradual and long-term reduction in body weight, and in particular a reduction in adipose tissue mass. Recently, monotrophic, unbalanced diets have become popular, and are considered the best methods of rapid weight loss. Scientific research indicates that long-term consumption of a high-fat diet has a negative effect on cardiometabolic risk. High-fat intake accompanied by restricted carbohydrate intake leads to changes in the hormonal balance, including low insulin concentration in the blood serum [14], which is also confirmed by our own research. Moreover, not only diets, but also single meals can induce significant adverse effects, called postprandial dysmetabolism, which is a postprandial state characterized by abnormal metabolism of glucose and lipids. Bell DSH. et al. demonstrated direct and proportional associations between postprandial dysmetabolism and increased inflammation, endothelial dysfunction, coronary artery disease as well as cardiac events [9]. There is evidence that postprandial dysmetabolism is associated with non-alcoholic fatty liver disease (NAFLD), increased cardiovascular mortality and morbidity, and some other diseases, mostly due to macro- and microvascular complications [15].

The current study demonstrated that a high-fat meal exerts an adverse effect on cardiometabolic risk factors (in particular, triglyceride and homocysteine concentrations), especially in overweight/obese individuals. Other authors have also observed an increase in serum triglyceride concentrations following a high-fat meal [16,17]. Furthermore, high-fat diets can significantly increase homocysteine concentrations, and thus have a detrimental effect on the function of the endothelium of blood vessels, particularly in people with excess body weight. Apart from affecting the lipid profile, impairment of endothelial function may be a possible mechanism in the pathophysiology of atherosclerosis from HF intake [18,19]. A study by Wycherley et al. also showed an increase in fasting homocysteine concentrations in individuals with excess body weight following consumption of a high-fat diet for one year [19]. However, not all studies have proven the effect of high-fat meals on homocysteine concentrations. In a study involving healthy adults, De Rose et al. did not find significant changes in serum homocysteine levels after HC or HF meal ingestion [20,21]. In the majority of scientific publications, high-protein diets were also the focus of interest in the context of the effect on homocysteine concentration [22].

Some authors suggest that changes observed in lipid levels depend on the duration of the diet. In the first six months, the changes may seem insignificant, while after a longer period, consumption of a high-fat diet may result in disturbances in lipid metabolism, mainly in the form of elevated levels of total cholesterol, triglycerides and serum homocysteine, thus increasing the risk of CVD. Changes in lipid concentrations seem to be particularly dangerous for people with obesity and dyslipidemia [20,21,22].

In the present study, postprandial serum glucose concentrations (followig HF and HC meals) were not statistically significantly correlated with BMI, which suggests that both overweight/obese and normal body weight individuals without carbohydrate metabolism disorders metabolize glucose similarly after HF and HC meal ingestion.

Our study demonstrated that consumption of a HF meal increased the number of potential risk factors for CVD (homocysteine and triglycerides) compared to a HC meal. Our research found that overweight/obesity, which is in itself a risk factor for CVD, contributes to an increase in the levels of other risk factors, such as the concentration of homocysteine and triglycerides [23]. We noted that in the 4 h postprandial period, glucose and insulin concentrations were significantly higher following the HC meal in comparison to the HF meal. Our study revealed that in overweight/obese men, a different metabolic response is evoked by the ingestion of both an HF meal and an HC meal in comparison to people with a healthy body weight consuming the same meals. It has been demonstrated that individuals with overweight/obesity, but without metabolic disorders, differ in their metabolic response to stimulation with dietary fat [21,24].

Analysis of the results obtained from indirect calorimetry measurements shows that the rates of energy substrate oxidation did not differ between the studied groups, except at 240 min following the HC meal, when higher glucose oxidation rates in subjects with overweight/obesity were noted. Comparable glucose oxidation following the consumption of both test meals observed in overweight/obese men may suggest the onset of metabolic flexibility disturbances. Resting metabolism and diet-induced thermogenesis were not significantly different between the studied groups. In the group of overweight/obese men, the HC meal caused higher energy expenditure, which may suggest that long-term consumption of a high-fat diet may result in weight gain due to its lower thermal effect.

The conclusions of our study encourage an extended follow-up of the body’s response to stimulation with different meals in order to better understand the long-term effects of different diets on cardiometabolic risk factors, especially because some scientific reports do not show any harmful effects of the long-term consumption of high-fat and low-carbohydrate diets on the cardiovascular system [25]. However, episodes of hypoglycemia after meals with a very high-carbohydrate content may significantly worsen metabolic control and increase the risk of developing cardiovascular diseases. Our study included men without carbohydrate metabolism disorders, but differences in the metabolic and hormonal responses following the ingestion of the test meals were observed between the men with normal body weight and those with overweight/obesity. Excess body weight may increase the risk of developing metabolic diseases, such as type 2 diabetes, dyslipidemia, atherosclerosis and metabolic syndrome. Hypertrophic adipocytes are also characterized by increased secretion of pro-inflammatory cytokines, which are considered potential risk factors for cardiovascular disease. Future research should focus on assessing the use of various monotrophic diets, and also traditional, unbalanced diets (in different populations of patients),and their impact on individual cardiovascular risk factors. Moreover, future research should also take into account nutrigenetic polymorphisms, which may also determine how the body metabolizes fats, proteins and carbohydrates.

We observed that following HF or HC meal ingestion, overweight/obese men metabolized the main nutrients differently than men with normal body weight, and postprandial insulin secretion was also different (even without any significant differences in glucose concentrations). Moreover, consumption of a HF meal increased the concentration of triglycerides and homocysteine in the blood serum. Both HF and HC diets may increase the risk of CVD, and therefore monotrophic diets should be avoided.

An important limitation of our study is a small sample size. The main reason for this was long observation time during the visits, which was approximately 7–8 h at a time. Another limitation of our study is assignment of eligible individuals to the groups only on the basis of their BMI index. An additional limitation of this study is assessment of only triglyceride concentrations in the blood serum of the participants, without taking into account the other parameters of lipid metabolism. Futher research in groups broken down by sex and age range, and the percentage of adipose tissue with an extension to the full lipid profile of the participants should be designed.

The value of this study is increased by the fact that the same subjects participated in the crossover study, and that it was based on standardized meals with the same energy value. Conducting further observations on a larger population is required and may lead to interesting conclusions that may be applicable in everyday clinical practice.

## 5. Conclusions

1. It was observed that following a high-fat or high-carbohydrate meal, men with excessive body weight metabolize the main nutrients differently than men with normal body weight, and postprandial insulin secretion is also different (even without any significant differences in glucose concentrations).
2. Overweight/obesity, which is in itself a risk factor for cardiovascular disease, contributes to an increase in the concentration of other risk factors such as the concentration of homocysteine and triglycerides, which is referred to as cardiometabolic risk.
3. Consumption of a high-fat meal increased the number of potential risk factors for cardiovascular diseases (homocysteine and triglycerides) compared to a high-carbohydrate meal.

## Figures and Tables

**Table 1:** Participant characteristics.

	G_S_ (*n* = 13)	G_C_ (*n* = 13)	*p*
Age (years)	37.42 ± 3.15	32.10 ± 3.12	0.2431
BMI (kg/m^2^)	32.36 ± 2.03	23.81 ± 0.21	0.0003 *

The results are presented as the mean values ± SE. BMI—body mass index; G_S_—overweight/obese men; G_C_—normal body weight men. * *p* < 0.05.

**Table 2:** Composition of standardized meals.

	HC Meal (Nutridrink Fat-Free) Meal Portion: 300 mL	HF Meal (Calogen) Meal Portion: 95 mL
**Energy value**	450 kcal	450 kcal
**Carbohydrates**	100.5 g	4.0 g
**% of energy**	89.3%	4%
**Mono-and disaccharides**	42.3 g	3.8 g
**Glucose**	1.0 g	0 g
**Fructose**	0 g	0 g
**Lactose**	<0.0075 g	0 g
**Maltose**	36.3 g	0 g
**Saccharose**	5.0 g	3.8 g
**Polysaccharides**	57.9 g	0 g
**Fats**	0 g	47.5 g
**% of energy**	0%	96%
**Saturated fat**	0g	5 g
**Vegetable fat**	0 g	42.5 g
**Milk fat**	0 g	0 g
**Monounsaturated fats**	0 g	28.9 g
**Polyunsaturated fats**	0 g	13.6 g
**Cholesterol**	0 mg	0 mg
**Protein**	12 g	0 g
**% of energy**	10.7%	0%
**% of energy**		
**Fiber**	0 g	0 g

HC meal—high-carbohydrate meal; HF meal—high-fat meal.

**Table 3:** Serum concentrations of the biochemical parameters in overweight/obese (G_S_) men and normal body weight (G_C_) men on fasting (time 0′) and after (time 30′–240′) high-fat (HF) and high-carbohydrate (HC) meal. (*AUC* (area under the curve)).

	**High-Fat Meal (HF)**											
	**Glucose (mg/dL)**			**Insulin (IU/mL)**			**Triglycerides (mg/dL)**			**Homocysteine (µmol/L)**		
**Time**	**G_S_**	**G_C_**	***p***	**G_S_**	**G_C_**	***p***	**G_S_**	**G_C_**	***p***	**G_S_**	**G_C_**	***p***
0′	98.4 ± 10.58	94.5 ± 8.98	0.6622	14.7 ± 14.36	8.2 ± 2.30	0.0812	122.0 ± 58.36	80.5 ± 35.92	0.0377	17.3 ± 7.73	15.7 ± 5.78	0.3172
30′	95.3 ± 10.37	92.7 ± 9.29	0.8772	21.3 ± 21.88	10.1 ± 3.89	0.0355	127.1 ± 65.81	81.3 ± 37.01	0.0256	16.9 ± 6.03	13.8 ± 2.53	0.0686
60′	88.6 ± 12.44	89.9 ± 7.92	0.5041	21.1 ± 18.12	9.6 ± 3.72	0.0024	126.3 ± 77.72	84.3 ± 34.47	0.0576	24.7 ± 14.01	14.4 ± 2.35	0.0292
120′	89.7 ± 9.90	89.7 ± 6.40	0.4399	17.6 ± 10.77	8.4 ± 2.13	0.0065	166.3 ± 97.78	109.9 ± 34.51	0.0158	21.5 ± 6.91	13.0 ± 2.60	0.0011
180′	91.6 ± 9.26	88.0 ± 5.78	0.4257	15.4 ± 11.45	8.1 ± 2.66	0.0159	205.9 ± 91.93	136.5 ± 48.25	0.0311	22.9 ± 14.81	14.6 ± 4.82	0.2701
240′	90.9 ± 7.18	89.3 ± 6.77	0.9794	12.4 ± 8.27	8.5 ± 3.28	0.2184	206.6 ± 91.53	148.5 ± 72.81	0.1508	23.2 ± 10.80	14.7 ± 2.88	0.0377
AUC	21658 ± 334	21,593 ± 442	0.7004	4171 ± 857	2113 ± 157	0.0040	39,867 ± 5453	26,685 ± 2713	0.0240	5370 ± 539	3437 ± 147	0.0040
	**High-Carbohydrate Meal (HC)**											
	**Glucose (mg/dL)**			**Insulin (IU/mL)**			**Triglycerides (mg/dL)**			**Homocysteine (µmol/L)**		
**Time**	**G_S_**	**G_C_**	***p***	**G_S_**	**G_C_**	***p***	**G_S_**	**G_C_**	***p***	**G_S_**	**G_C_**	***p***
0′	96.2 ± 9.18	91.6 ± 18.31	0.6254	14.8 ± 14.10	10.2 ± 5.20	0.6628	113.5 ± 41.25	75.3 ± 41.03	0.0294	16.8 ± 4.17	13.2 ± 2.49	0.0221
30′	158.0 ± 25.65	152.3 ± 30.28	0.9794	181.1 ± 283.38	86.2 ± 31.06	0.7778	116.6 ± 39.65	77.0 ± 42.18	0.0294	19.4 ± 9.10	13.2 ± 4.02	0.0042
60′	169.3 ± 46.81	149.3 ± 31.33	0.3166	238.1 ± 342.86	120.8 ± 86.53	0.9795	118.3 ± 47.64	74.5 ± 42.35	0.0190	15.7 ± 7.35	13.8 ± 5.55	0.3107
120′	140.6 ± 51.61	127.0 ± 26.94	0.6440	141.1 ± 197.22	84.1 ± 41.21	0.7388	105.5 ± 45.08	62.9 ± 38.55	0.0119	19.0 ± 11.41	12.3 ± 1.74	0.0255
180′	105.3 ± 19.81	104.6 ± 25.86	0.8174	42.4 ± 33.24	45.9 ± 32.18	0.6628	102.8 ± 49.75	63.0 ± 42.15	0.0190	19.6 ± 11.12	12.6 ± 3.20	0.0190
240′	78.7 ± 19.00	77.1 ± 18.98	0.9386	17.1 ± 13.30	13.6 ± 6.02	0.8979	98.7 ± 51.86	71.5 ± 51.03	0.0908	15.1 ± 8.97	12.4 ± 3.27	0.5787
**AUC**	30,482 ± 1426	28,882 ± 1322	0.5726	27,907 ± 9545	16,396 ± 1784	0.8979	25,995 ± 3046	16,502 ± 2772	0.0274	4323 ± 511	3090 ± 141	0.0138

**Table 4:** Energy expenditure (kcal/min), glucose and fat oxidation (mg/min) in G_S_ (overweight/obesity) group and G_C_ (normal body weight) group on fasting (time 0′) and after (time 60′–240′) a high-fat (HF) meal and a high-carbohydrate (HC) meal. (*AUC* (area under the curve)).

	**High-Fat Meal (HF)**								
	**Energy Expenditure (kcal/min)**			**Glucose Oxidation (mg/min)**			**Fat Oxidation (mg/min)**		
**Time**	**G_S_**	**G_C_**	***p***	**G_S_**	**G_C_**	***p***	**G_S_**	**G_C_**	***p***
0′	1.332 ± 0.25	1.267 ± 0.24	0.3970	41.3 ± 47.76	34.0 ± 43.71	0.5552	71.4 ± 36.46	56.4 ± 33.22	0.2811
60′	1.333 ± 0.27	1.263 ± 0.19	0.6628	61.4 ± 66.25	34.4 ± 45.07	0.2084	82.4 ± 48.55	87.3 ± 31.98	0.8979
120′	1.335 ± 0.23	1.269 ± 0.22	0.2181	43.9 ± 48.04	34.3 ± 40.93	0.6628	91.5 ± 40.76	90.1 ± 29.02	0.9590
180′	1.336 ± 0.26	1.217 ± 0.10	0.1237	24.9 ± 19.29	27.6 ± 41.69	0.5724	103.0 ± 32.43	92.0 ± 27.96	0.3426
240′	1.339 ± 0.24	1.327 ± 0.20	0.2928	23.5 ± 9.66	31.9 ± 45.45	0.5046	105.5 ± 32.80	94.4 ± 30.18	0.4263
AUC	325.64 ± 16.478	302.87 ± 9.526	0.3693	9775 ± 1987	7765 ± 2738	0.2088	21,936 ± 2305	20,699 ± 1937	1.0000
	**High-Carbohydrate Meal (HC)**								
	**Energy Expenditure (kcal/min)**			**Glucose Oxidation (mg/min)**			**Fat Oxidation (mg/min)**		
**Time**	**G_S_**	**G_C_**	***p***	**G_S_**	**G_C_**	***p***	**G_S_**	**G_C_**	***p***
0′	1.329 ± 0.22	1.222 ± 0.15	0.1369	23.9 ± 22.60	28.6 ± 22.5	0.4265	87.7 ± 28.40	66.2 ± 26.50	0.1061
60′	1.447 ± 0.30	1.338 ± 0.09	0.5381	46.0 ± 55.44	46.7 ± 56.01	0.9386	93.4 ± 34.56	77.7 ± 23.98	0.1822
120′	1.395 ± 0.29	1.311 ± 0.10	0.7583	68.7 ± 60.23	76.2 ± 49.84	0.3692	78.5 ± 28.98	63.6 ± 22.98	0.1689
180′	1.325 ± 0.26	1.220 ± 0.14	0.6816	62.9 ± 63.27	67.6 ± 53.88	0.3972	71.7 ± 26.32	57.5 ± 24.06	0.1689
240′	1.316 ± 0.29	1.240 ± 0.12	0.7583	42.5 ± 30.08	35.0 ± 46.29	0.0479	84.7 ± 36.40	77.6 ± 22.67	0.6138
**AUC**	329.48 ± 18.328	306.14 ± 7.191	0.6444	12,665 ± 3013	13,351 ± 2689	0.3558	19,799 ± 1901	16,253 ± 1322	0.1909

## Data Availability

Data available on request due to restrictions e.g., privacy or ethical.

## Supplementary Materials

The following supporting information can be downloaded at: https://www.mdpi.com/article/10.3390/nu14142876/s1, Figure S1: (a) Serum glucose concentration (mg/dL) in the G_S_ and G_C_ groups fasting (time 0′) and after intake (time 30′–240′) of HF meal, (b) Serum glucose concentration (mg/dL) in the G_S_ and G_C_ groups fasting (time 0′) and after intake (time 30′–240′) of HC meal; Figure S2: (a) Serum insulin concentration (IU/mL) in the G_S_ and G_C_ groups fasting (time 0′) and after intake (time 30′–240′) of HF meal, (b) Serum insulin concentration (IU/mL) in the G_S_ and G_C_ groups fasting (time 0′) and after intake (time 30′–240′) of HC meal; Figure S3: (a) Serum triglycerides concentration (mg/dL) in the G_S_ and G_C_ groups fasting (time 0′) and after intake (time 30′–240′) of HF meal, (b) Serum triglycerides concentration (mg/dL) in the G_S_ and G_C_ groups fasting (time 0′) and after intake (time 30′–240′) of HC meal; Figure S4: (a) Serum homocysteine concentration (µmol/l) in the G_S_ and G_C_ groups fasting (time 0′) and after intake (time 30′–240′) of HF meal, (b) Serum homocysteine concentration (µmol/L) in the G_S_ and G_C_ groups fasting (time 0′) and after intake (time 30′–240′) of HC meal; Figure S5: (a) Energy expenditure (kcal/min) in the G_S_ and G_C_ groups fasting (time 0′) and after intake (time 60′–240′) of HF meal, (b) Energy expenditure (kcal/min) in the G_S_ and G_C_ groups fasting (time 0′) and after intake (time 60′–240′) of HC meal; Figure S6: (a) Glucose oxidation (mg/min) in the G_S_ and G_C_ groups fasting (time 0′) and after intake (time 60′–240′) of HF meal, (b) Glucose oxidation (mg/min) in the G_S_ and G_C_ groups fasting (time 0′) and after intake (time 60′–240′) of HC meal; Figure S7: (a) Lipid oxidation (mg/min) in the G_S_ and G_C_ groups fasting (time 0′) and after intake (time 60′–240′) of HF meal, (b) Lipid oxidation (mg/min) in the G_S_ and G_C_ groups fasting (time 0′) and after intake (time 60′–240′) of HC meal.
