# Supplementary material for: High-Fat or High-Carbohydrate Meal—Does It Affect the Metabolism of Men with Excess Body Weight?
Source: Nutrients. 2022 Jul 13;14(14):2876. doi: 10.3390/nu14142876 (PMC9323987; doi:10.3390/nu14142876)
Supplement: Supplementary file 1 [file nutrients-14-02876-s001.zip › nutrients-1785695-supplementary.pdf]

Supplementary Files

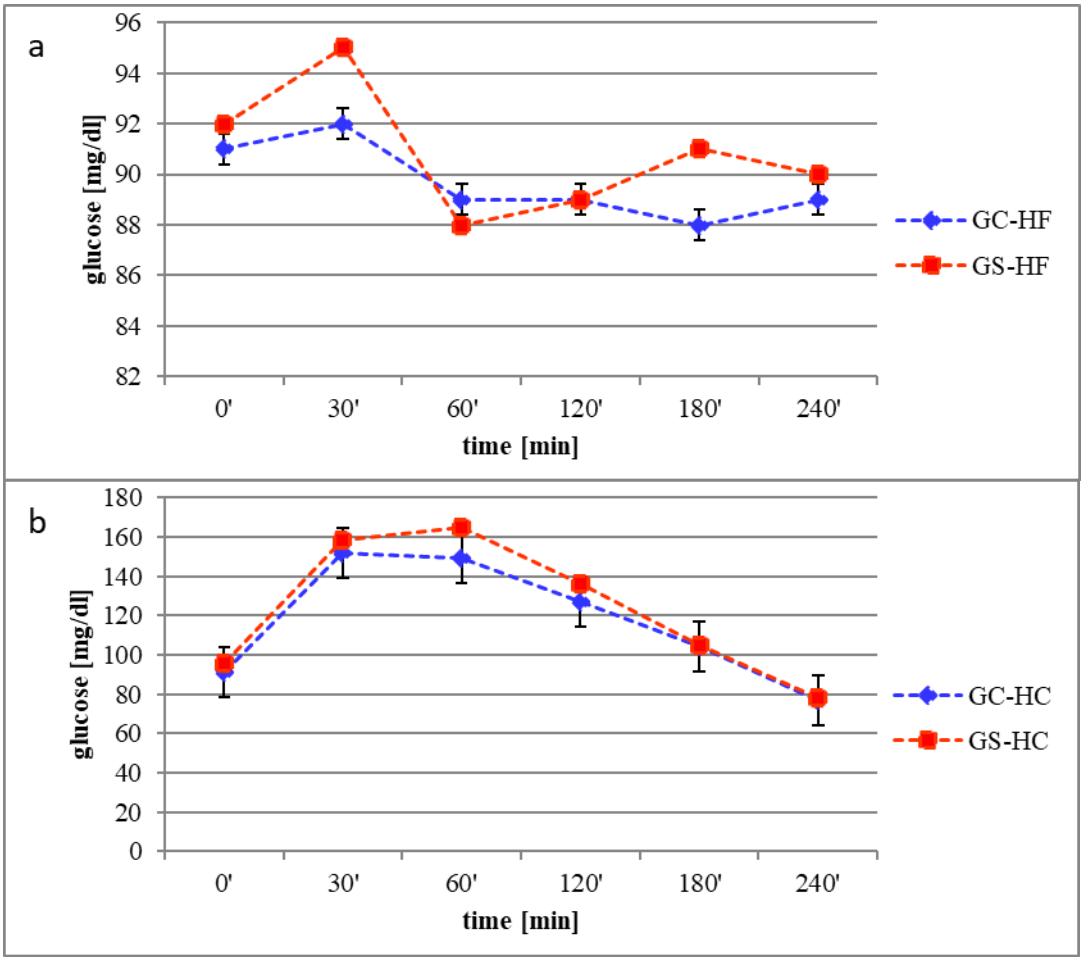

Figure S1. (**a**) Serum glucose concentration (mg/dl) in the GS and GC groups fasting (time 0′) and after intake (time 30′-240′) of HF meal. (**b**) Serum glucose concentration (mg/dl) in the GS and GC groups fasting (time 0′) and after intake (time 30′-240′) of HC meal.

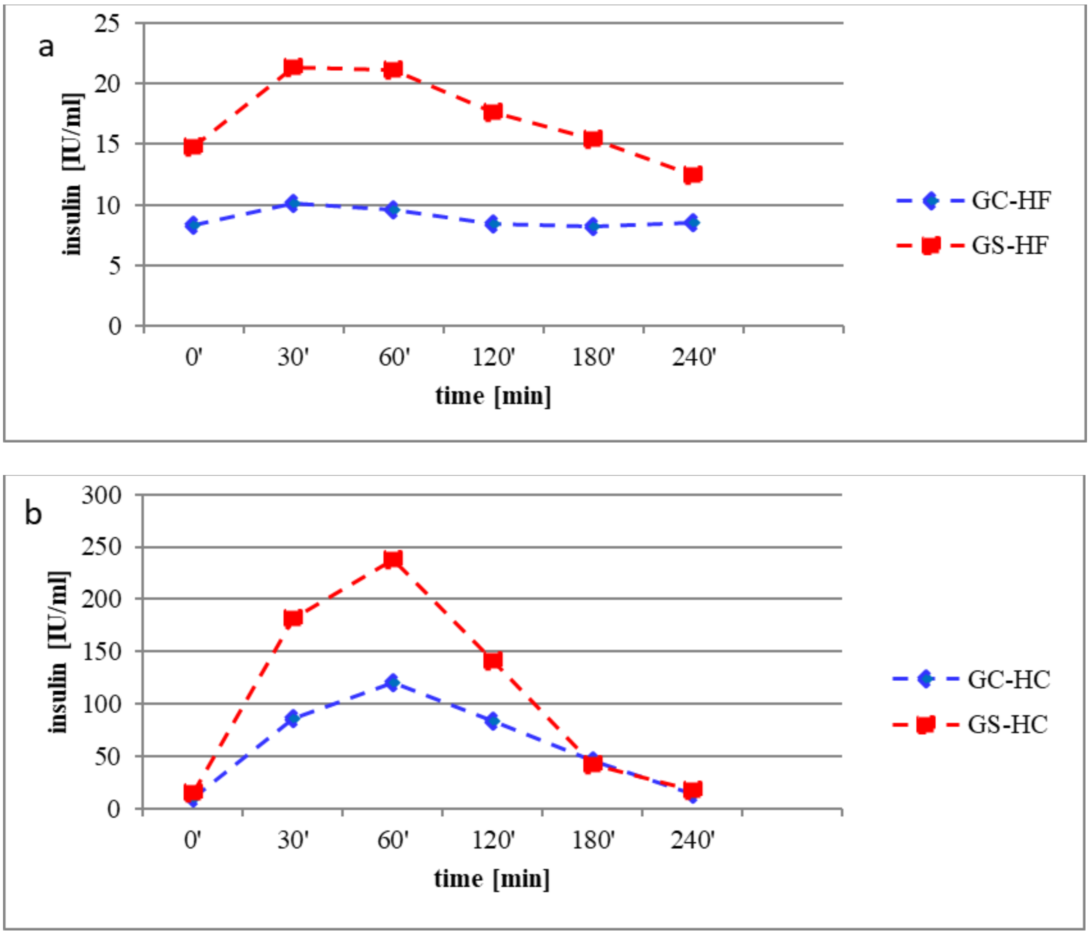

Figure S2. (**a**) Serum insulin concentration (IU/ml) in the G_S and G_C groups fasting (time 0′) and after intake (time 30′-240′) of HF meal. (**b**) Serum insulin concentration (IU/ml) in the G_S and G_C groups fasting (time 0′) and after intake (time 30′-240′) of HC meal.

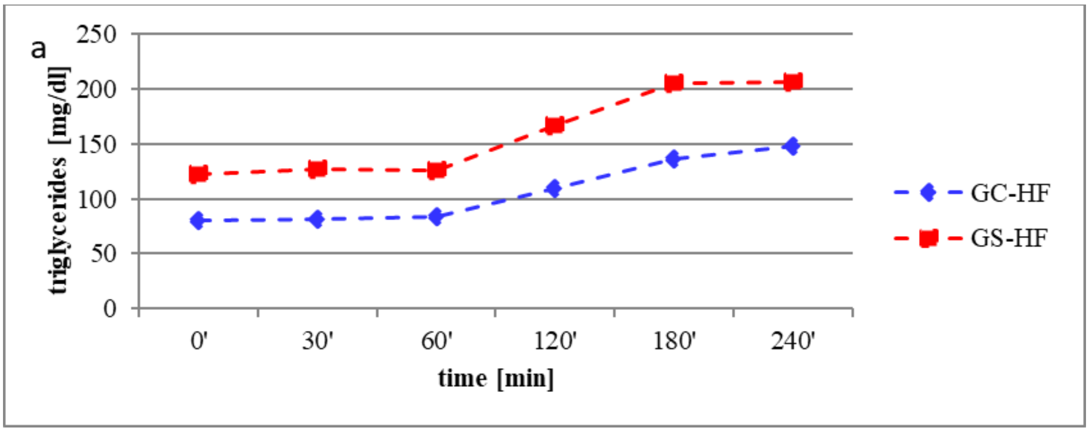

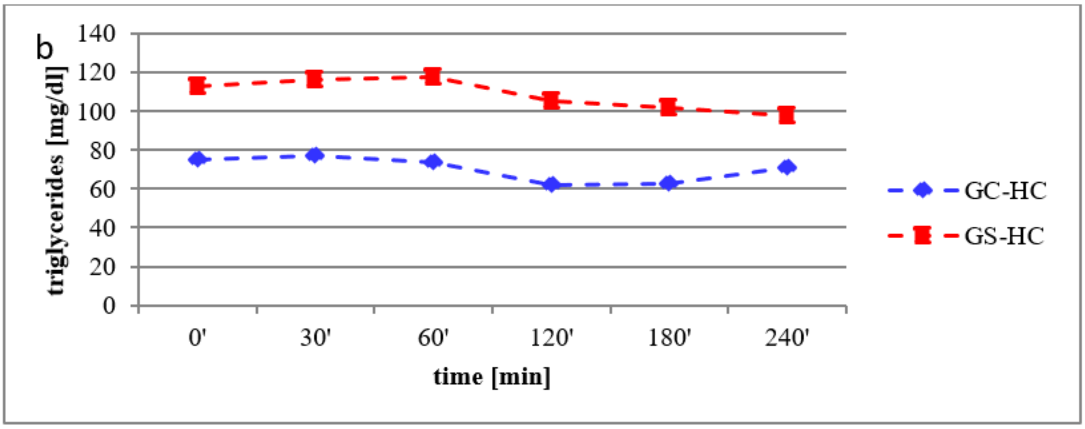

Figure S3. (**a**) Serum triglycerides concentration (mg/dl) in the Gs and Gc groups fasting (time 0′) and after intake (time 30′-240′) of HF meal. (**b**) Serum triglycerides concentration (mg/dl) in the Gs and Gc groups fasting (time 0′) and after intake (time 30′-240′) of HC meal.

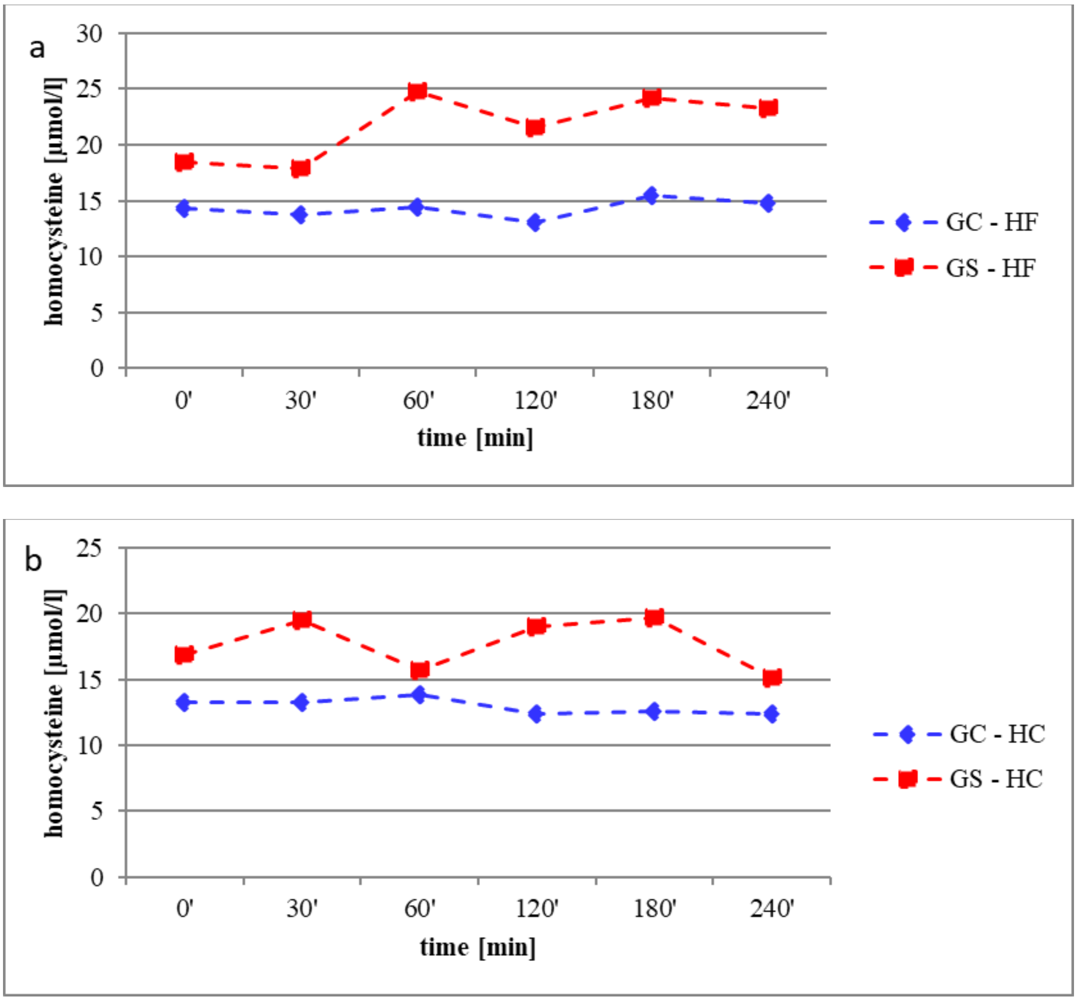

Figure S4. (**a**) Serum homocysteine concentration (µmol/l) in the G_S and G_C groups fasting (time 0′) and after intake (time 30′-240′) of HF meal. (**b**) Serum homocysteine concentration (µmol/l) in the G_S and G_C groups fasting (time 0′) and after intake (time 30′-240′) of HC meal. .

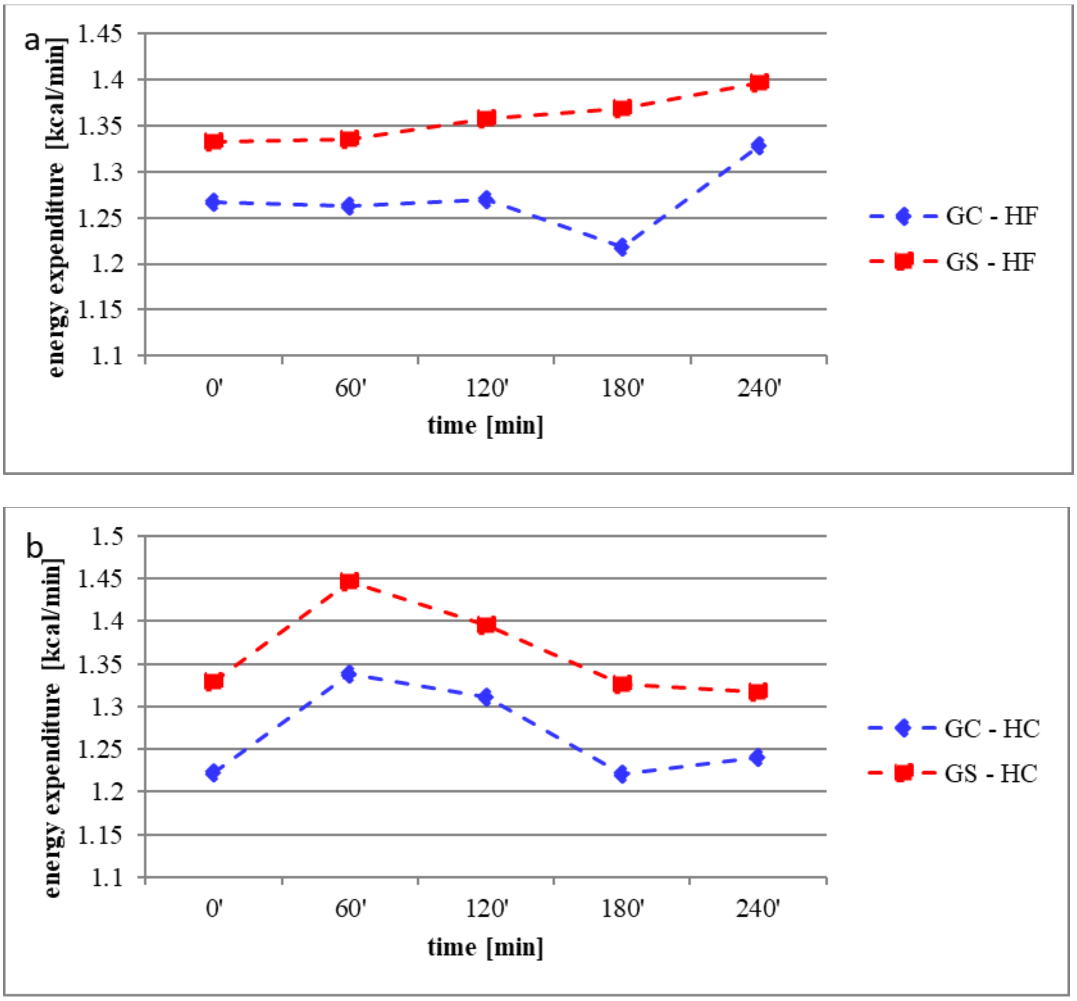

Figure S5. (**a**) Energy expenditure (kcal/min) in the G_S and G_C groups fasting (time 0′) and after intake (time 60′-240′) of HF meal. (**b**) Energy expenditure (kcal/min) in the G_S and G_C groups fasting (time 0′) and after intake (time 60′-240′) of HC meal.

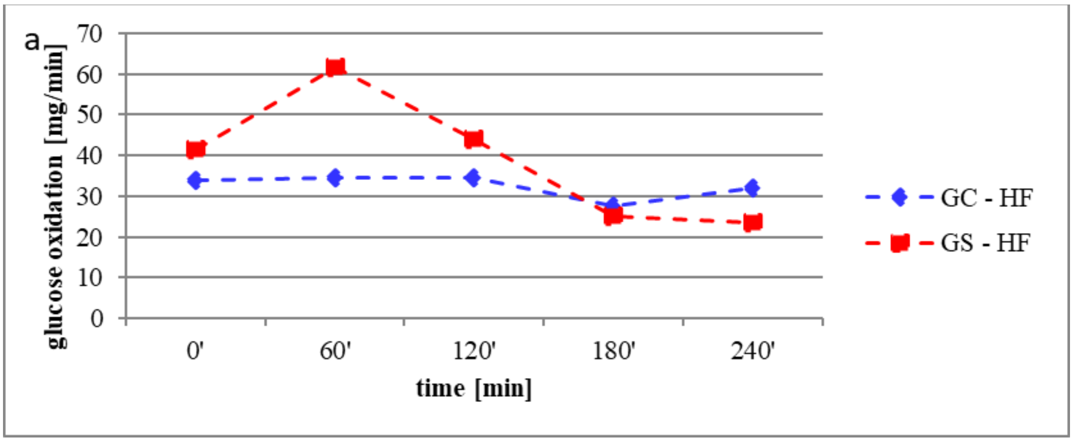

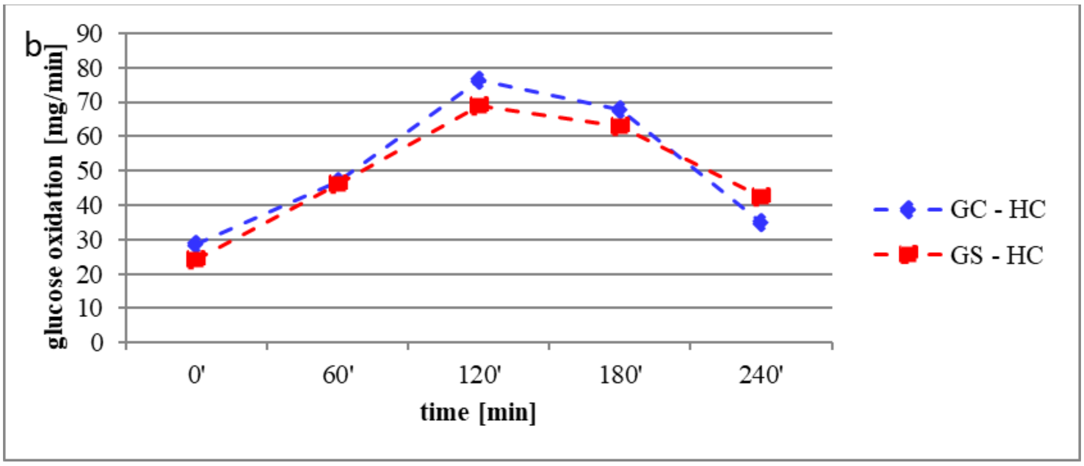

Figure S6. (**a**) Glucose oxidation (mg/min) in the Gs and Gc groups fasting (time 0′) and after intake (time 60′-240′) of HF meal. (**b**) Glucose oxidation (mg/min) in the Gs and Gc groups fasting (time 0′) and after intake (time 60′-240′) of HC meal.

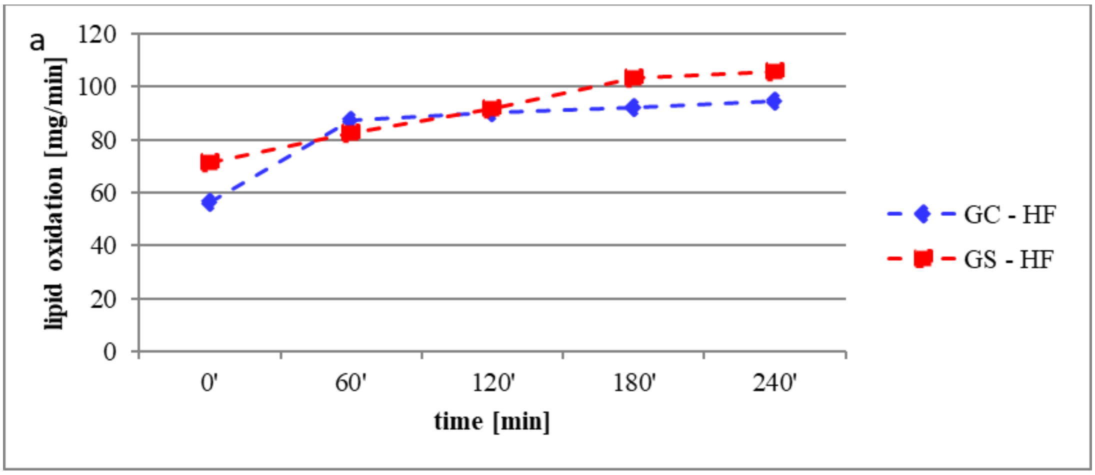

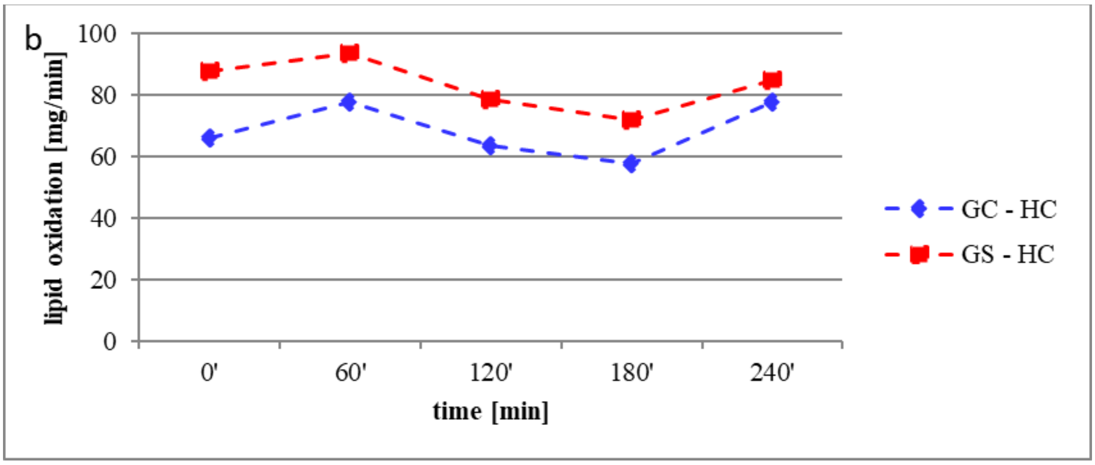

Figure S7. (**a**) Lipid oxidation (mg/min) in the Gs and Gc groups fasting (time 0′) and after intake (time 60′-240′) of HF meal. (**b**) Lipid oxidation (mg/min) in the Gs and Gc groups fasting (time 0′) and after intake (time 60′-240′) of HC meal.
